# Causal Illusions in the Service of Political Attitudes in Spain and the United Kingdom

**DOI:** 10.3389/fpsyg.2018.01033

**Published:** 2018-06-28

**Authors:** Fernando Blanco, Braulio Gómez-Fortes, Helena Matute

**Affiliations:** ^1^Departamento de Fundamentos y Métodos de la Psicología, University of Deusto, Bilbao, Spain; ^2^Facultad de Ciencias Sociales y Humanas, University of Deusto, Bilbao, Spain

**Keywords:** cognitive bias, causal illusion, political orientation, motivated reasoning, causality

## Abstract

The causal illusion is a cognitive bias that results in the perception of causality where there is no supporting evidence. We show that people selectively exhibit the bias, especially in those situations where it favors their current worldview as revealed by their political orientation. In our two experiments (one conducted in Spain and one conducted in the United Kingdom), participants who self-positioned themselves on the ideological left formed the illusion that a left-wing ruling party was more successful in improving city indicators than a right-wing party, while participants on the ideological right tended to show the opposite pattern. In sum, despite the fact that the same information was presented to all participants, people developed the causal illusion bias selectively, providing very different interpretations that aligned with their previous attitudes. This result occurs in situations where participants inspect the relationship between the government’s actions and positive outcomes (improving city indicators) but not when the outcomes are negative (worsening city indicators).

## Introduction

The social and physical worlds in which people live are in constant change. This means that people need to continuously adapt to make good decisions and hold accurate beliefs: what used to be right in the past can be utterly wrong today. One of the key abilities that allow such adaptation of behavior is causal learning because it enables people to associate causes and effects on the light of extant evidence and experience (Holyoak and Cheng, 2011). However, this goal of holding accurate, updated beliefs contrasts with people’s need to keep a consistent worldview that is not constantly contradicted by noisy or unreliable evidence. Indeed, people do not like to change their minds about important issues on a daily basis. There is a tension between the tendency to adaptation and change and the need of protection for important beliefs. In addition, it is parsimonious to propose that the same cognitive system that learns about the changes in the environment is also permeable to preexisting beliefs and ideology, so that these latter can be protected from harm (Bramley et al., 2015).

One novel way in which causal learning can be modeled to inadvertently achieve this alignment with previous beliefs is via cognitive biases. A cognitive bias is revealed as a systematic error or deviation from rationality in judgment or decision-making (Haselton et al., 2005). Importantly, cognitive biases of many types have been described in the general population: they are not reduced to pathological or handicapped reasoning. Rather, they are usually considered adaptive, as they can lead to sensible-enough and fast decisions. One important feature of cognitive biases is that they can be used to reject conflicting information or to magnify data that support the current beliefs. For example, the literature on motivated reasoning shows that people actually reason in ways that favor their prior political attitudes (Kunda, 1990), which is akin to showing a confirmation bias (Oswald and Grosjean, 2004). In a classical experiment, researchers presented participants with two scientific studies on the effectiveness of death penalty to reduce crime rate (Lord et al., 1979). According to one of the studies, death penalty lowered the crime rate, whereas the other study supported the opposite conclusion. Additionally, the researchers gave participants information about the potential shortcomings and methodological flaws of both studies. That is, the studies contributed ambiguous information: although they claimed to support one hypothesis, their arguments were imperfect. Lord et al. (1979) reported that participants in fact gave higher credibility ratings to the study that supported their own pre-existing view on death penalty, and pointed the problems in the study that contradicted such view, even when both studies were methodologically identical. Thus, the confirmation bias can affect the way people treat scientific evidence (e.g., neglecting it when it collides with such existing attitudes) (Bastardi et al., 2011). A similar process could take place when learning causal relationships from evidence.

How can people accurately learn whether a causal relationship exists? It is traditionally assumed that this learning is strongly based on the detection of correlations, or contingencies, between potential causes and effects (Perales and Shanks, 2007). The rationale for this relationship between contingency and causation derives from the fact that, unless a confounding factor is playing a role, every event is contingent on its own causes (Hume, 1748). Thus, as causes and effects are expected to correlate, detecting these relationships, or contingencies, is a valuable skill to infer potential causality. In line with this reasoning, a great corpus of experimental evidence strongly suggests that manipulations of the contingency between potential causes and effects produce the corresponding variations in people’s judgments of causality (Allan and Jenkins, 1980; Shanks and Dickinson, 1987; Dodwell and Humphrey, 1993; Blanco et al., 2010).

**FIGURE 1:**
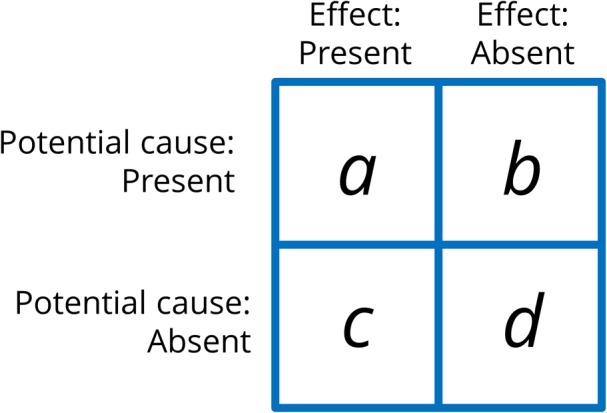
Contingency matrix containing the four types of trials used in a contingency learning experiment (*a*, *b*, *c*, and *d*). The actual contingency or correlation between cause and effect can be computed from the frequencies of each of these trials types.

In a typical contingency learning experiment, participants are presented with a sequence of several trials of four types: sometimes, the potential cause is followed by the effect (type *a* trial), whereas sometimes it is not (type *b* trial), some other times, the effect appears without the potential cause being present (type *c* trial), and finally, there are times when neither the potential cause nor the effect occurs (type *d* trial) (see **Figure 1**). The amount of trials of each type can be manipulated to obtain trial sequences that vary in their degree of contingency. This contingency can be determined objectively by rules such as ΔP (Allan and Jenkins, 1980, see Equation 1):

$$\Delta P=P(effect|cause)-P(effect|\sim cause)=\frac{a}{a+b}-\frac{c}{c+d}\,$$

This index expresses the degree of contingency between cause and effect, and is computed from the frequency of each trial type (*a*, *b*, *c*, and *d*, see **Figure 1**). A value of zero indicates that cause and effect are not correlated to each other. The more contingency departs from zero, the stronger the relationship between the cause and the effect. Thus, it is possible to create materials that show any degree of contingency by manipulating the trial frequencies. Additionally, observe that we can obtain low or even zero contingency values while keeping the rate of occurrences of the effect and the cause high (what matters in a zero contingency setting is that the proportion of effect occurrences is the same regardless of whether the cause is present or not).

As we have mentioned, many experiments have shown that people are sensitive to these variations in contingency. However, experiments using similar procedures have also showed that causal judgments can be easily biased by certain factors. For instance, when the experimenters hold the contingency between a potential cause and its potential effect fixed to zero (which means that the two stimuli do not correlate and, consequently, that no causal relationship should be inferred), presenting many cases in which the potential cause and the potential effect coincide (e.g., many type *a* trials) leads to a systematic overestimation of the causal relationship. This result is usually known as “causal illusion” (Matute et al., 2015), a cognitive bias in causal learning that consists of people believing that two events are causally related even when the objective information that is presented during the experiment indicates that they are perfectly uncorrelated. This bias is particularly strong when the potential effect occurs with high probability (Alloy and Abramson, 1979; Allan and Jenkins, 1983; Buehner et al., 2003), or when the potential cause occurs with high probability (Perales et al., 2005; Hannah and Beneteau, 2009; Blanco et al., 2011). Null contingencies accompanied by a high probability of the potential cause and/or of the potential effect can convey ambiguous information because the two stimuli will likely co-occur often even if just by chance. This is true despite the objective correlation between the potential cause and effect being low or zero. Causal illusions are known to be stronger in these cases, that is, people perceive the potential cause as causing the effect if the two events occur together frequently, by chance.

The causal illusion and related biases have been proposed to underlie many societal problems including social prejudice and stereotype formation (Hamilton and Gifford, 1976; Kutzner et al., 2011; Matute et al., 2015). Importantly, the causal illusion has been detected in healthy participants (it is not the result of a disordered or ill-adapted mind), and it even shows variations depending on factors that affect the general population, such as mood (Alloy and Abramson, 1979; Msetfi et al., 2005; Blanco et al., 2012). In general, this illusion is considered a basic feature of the way normal cognitive systems work, rather than an anomaly. Therefore, the illusion of causality can reveal much about our cognition.

Our current proposal is that causal illusions could play a relevant role in the way people deal with the information that collides with their preexisting worldviews. As it is a feature that appears in the general population and shows variations in response to personal and situational factors (e.g., mood, frequency of the effect), it would endow people with the flexibility to accommodate conflicting information and previous knowledge. In previous studies, people made systematic errors when reasoning about causes and effects, so that their personal conclusions fit with their preexisting political worldview (Kahan et al., 2012), in a way that resembles the confirmation bias. In those experiments, the data used for causal inference were presented to participants in tables summarizing the contingency information, which clearly favored or threatened the participant’s pre-experimental views on a political issue (i.e., gun control). Then, people systematically biased their estimation of the contingency between a potential cause (banning carrying concealed handguns in public) and an effect (increase in crime rate), and the direction of the bias was predicted by their prior beliefs on the topic: supporters of gun rights tended to overestimate a slightly negative contingency between the adoption of gun control laws and the increase in crime rate and showed the exactly opposite pattern when the data in the contingency table were rearranged to produce a slightly positive contingency.

As we mentioned, this study on “motivated numeracy” (Kahan et al., 2012) suggests that the perception of the contingency contained in a table can be affected by previous beliefs. Importantly, however, contingency tables are not an ecologically valid model of how people face changing contingencies in their everyday life, so the extension of these results to causal learning in general is not warranted. Furthermore, researchers have recurrently shown that the presentation format of the contingency information is a highly relevant factor in causal learning (White, 2003; Vallée-Tourangeau et al., 2008). Instead of arranging the information in a contingency table, the so-called ‘trial-by-trial’ procedures present separate pieces of information sequentially. These procedures are closer to the way people usually find and treat relevant information in the real world (i.e., scattered instead of conveniently arranged). Thus, trial-wise presentation of data in the laboratory better models how people gradually modify their judgments in natural settings (Wasserman, 1990; Shanks, 1991). Not surprisingly, most of the research in causal illusions has used the trial-by-trial procedure (Matute et al., 2015).

Some recent experiments have used more ecologically valid trial-by-trial procedures to address the question of whether causal illusions are sensitive to prior beliefs. These studies suggested that, in fact, people bias their causal learning so that it aligns with their previous knowledge (Yarritu and Matute, 2015). However, in these studies, the previous knowledge concerned issues such as fictitious medicines and diseases that were studied during the experiment and involved no connections to the participants’ belief system. Thus, it is unclear how people would face materials that occur in a trial-by-trial progression and that either concur or conflict with their beliefs and worldview, beyond a merely artificial and fictitious setup. A reasonable prediction, based on previous experiments and consistent with the motivated reasoning accounts (Kunda, 1990; Kahan et al., 2012), is that people would take advantage of the ambivalence of the experimental materials (i.e., they can be interpreted either as favoring or refuting a conclusion) to reach the causal conclusions that fit better with their existing ideology and political views. According to this rationale, people would develop the causal illusion selectively to protect their pre-existing beliefs. Hence, the occurrence and magnitude of causal illusions will depend on the individual participant’s ideology and on the content and implications of the to-be-learned materials. The present research was aimed at testing this prediction.

Additionally, there are different potential ways in which people could use the causal illusion to protect their previous beliefs. First, they could strengthen the illusion when it is framed in favorable terms that align with previous beliefs (e.g., a person who identifies with Group A would develop the illusion that Group A is able to produce a positive effect or outcome). Alternatively, they could strengthen the illusion when it is framed in unfavorable ways that also align with previous beliefs (e.g., a person who holds a negative belief against Group B would develop the illusion that Group B is able to produce a negative effect or outcome). These are not incompatible behaviors, as people could do both things at the same time. To explore this possibility, we included another manipulation in our experiments, outcome valence (positive/negative). That is, half of our participants were exposed to a situation where potential causal relationship was framed in positive terms, whereas for the other half the situation was framed in negative terms. Most research in causal illusions has used positive outcomes (but see Matute and Blanco, 2014; Blanco and Matute, 2015), although the rationale presented earlier should in principle apply also to negative outcomes, thus reversing the predicted results in this condition. The outcome-valence manipulation allows us to explore potential differences in the magnitude of the effect between the two types of scenario: perhaps the previous-belief impact on the causal illusion would be stronger when it is driven by a positive, compared to a negative, belief, but it could be the opposite, or even they could be similarly strong (but note that we always predict that the effects in positive- vs. negative-valenced outcomes should take opposite directions, regardless of magnitude).

## Experiment 1

### Method

#### Participants and Apparatus

The sample size was decided after examining the results of a previous study (not published) with conceptually identical goals. In this previous study (*N* = 56), the crucial effect (the interaction between the framing of the cover-story and ideology on the causal judgments) was *R*^2^ = 0.1632. To observe such an effect with power = 0.80, we needed a sample of between 34 and 43 participants (all calculations were conducted with the software G^∗^Power 3, Faul et al., 2007). Given that the design of our current experiment features two groups to explore a new manipulation (outcome valence), we decided to double this required sample size.

In total, 88 participants (out of which 60 were women) visited the website of our laboratory. They took part in the study in exchange for the opportunity to win a prize of 100 € in a raffle. Instead of stopping data collection when we reached the desired sample size, we determined to continue collecting participants online until the raffle was carried out (the exact date of the raffle was made public to all potential participants before collecting the sample). A sensitivity analysis reveals that with this sample size and design, we can detect effects as small as *f* = 0.302 with 80% power.

The experiment was a *javaScript* program embedded in an HTML document and styled by CSS. The program randomly assigned participants to the two groups, resulting in 42 participants in the Negative Outcome group and 46 in in the Positive Outcome group. All participants were adults (mean age of 26.82 years, *SD* = 12.64) and completed primary education. Their education level was distributed as indicated in **Table 1**.

**Table 1:** Distribution of participants per education level (Experiment 1).

Education level	*n*
Secondary education	36
Vocational training	17
University studies	22
Postgraduate studies	13


#### Ethics Statement

The procedure used in these experiments was examined and approved by the Ethical Review Board of the University of Deusto (Ref: ETK-12/14-15). The participants were informed before the experiment that they could quit the study at any moment by closing the browser window. The data collected during the experiment were sent anonymously to the experimenter only upon explicit permission by the participant, indicated by clicking on a “Submit” button. If the participant clicked on the “Cancel” button, the information was erased. No personal information (i.e., name, IP address, e-mail) was collected. We did not use cookies or other means to covertly obtain information from the participants. All data and experiment materials are publicly available at the Open Science Framework (Blanco et al., 2018).

#### Procedure

We adapted the standard trial-by-trial contingency learning task (Wasserman, 1990) that has been extensively used to study causal illusions (Matute et al., 2015). In this experiment, participants were exposed sequentially to two scenarios or causal stories that were meaningful to their personal worldviews. Specifically, they were instructed to play the role of a journalist who was examining the effectiveness of the actions taken by the government of an imaginary country. To do so, they would know whether a given action proposed by the government was eventually carried out or not. Additionally, they would know whether or not the application of the action was followed by improvements in success indicators. It was explicitly suggested that, if the actions are good, then the indicators would improve, but if the actions are bad, then they would get worse.

**FIGURE 2:**
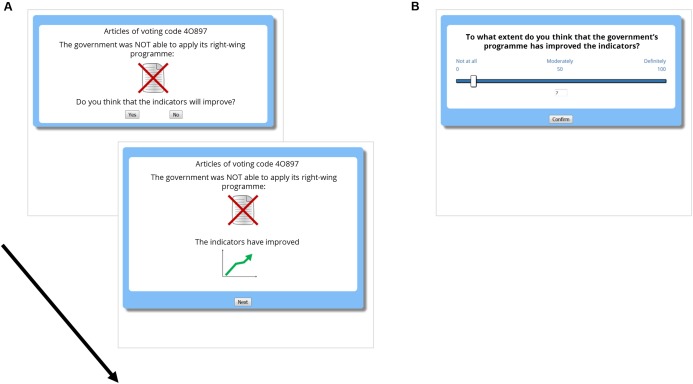
Experimental task. **(A)** Depicts two moments within the same trial: First, the information about the potential cause is presented (in this example, the government, which is right-wing, did not apply its program, i.e., the cause was not present). Once the prediction is made, the outcome is presented (in this example, the city indicators improved, i.e., a positive outcome was presented). **(B)** Depicts the screen where judgments were collected after each phase (40 trials).

Thus, participants were exposed to two series of records (trials) that showed information about the actions taken by the government and about the success indicators. The series were presented sequentially, in different phases of the experiment (Phase 1 and Phase 2), each of them consisting of 40 trials (see **Figure 2A**). On each trial, the information about the political action was presented at the top of the screen: either the government approved or not approved the action. Note that the procedure did not specify which action in particular was being considered in each trial. Rather, the actions (potential causes) were always formulated in vague terms, as the screenshot shown in **Figure 2A**. This forced participants to consider the actions taken by the government (the potential cause) globally, considering all trials, with no action (i.e., potential cause in a given trial) having systematically more weight than others. Then, participants answered whether or not they thought that the indicators would change (by clicking on a “Yes/No” button). This trial-by-trial prediction is usually included in contingency learning procedures because it helps participants to stay focused on the task. Immediately after making the prediction, they were told about whether the indicators actually changed (**Figure 2A**, lower panel): either the indicators improved (in the Positive Outcome group)/worsened (Negative Outcome group) or did not change. After clicking on a button labeled “Next,” a new trial started. There was no time-limit for progressing through the task (participants advanced at their own pace). After each series of 40 trials, participants were asked to judge to what extent the actions taken by the government had an impact on the success indicators (**Figure 2B**), on a scale from 0 (nothing at all) to 50 (moderately) to 100 (totally). These final judgments were our dependent variable.

In sum, participants were asked to examine the potential relationship between the government taking actions (potential cause) and the changing indicators (potential outcome). The contingency between the potential cause and the outcome was always zero: The probability of change when the action was taken was 0.8, the same as when the action was not taken (**Table 2** contains the frequencies of each type of trial, which were presented in random order). That is, there was absolutely no statistical connection between the government’s actions and the changes in the indicators, as computed by Equation 1. Still, because there were many more outcome-present trials than outcome-absent trials (32 vs. 8), we expected that some participants would develop an illusion of causality, in line with previous studies. Note that, since the actual contingency is zero, any judgment greater than zero would represent some degree of overestimation of causality, but the greater the judgment, the stronger the bias.

**Table 2:** Frequencies of each type of trial in each training phase (40 trials).

	Outcome-present	Outcome-absent
Cause-present	16 trials	4 trials
Cause-absent	16 trials	4 trials


The two consecutive phases (or series of 40 trials) differed in howns. One of the phases concerned the actions taken by a left-wing party, whereas the other concerned a right-wing party. This means that, depending on their political orientation, some participants would align themselves with the party in charge in one of the series of trials, but not with the other. The order of the two phases was randomly selected by the computer. Except for this instructional framing difference, the two phases consisted of 40 trials conveying exactly the same contingency information (**Table 2**).

Moreover, as advanced in the Introduction, we included an additional manipulation. Half of participants were assigned to the Positive Outcome group in which they had to pay attention to whether indicators improved. In the Negative Outcome group, they were asked to determine if the indicators got worse. This outcome valence manipulation was included to test the possibility that the bias was driven specifically by the desire of seeing the preferred group to succeed, or by the desire of seeing the opposite group to fail. In principle, we have no reasons to expect asymmetries between these two groups, and consequently we predict that the effect of previous beliefs on the judgments would be reversed when the outcome is negative (i.e., symmetrical results in the two groups). However, it could be the case that people prefer to bias their judgments more in the positive than in the negative scenario, or vice versa. **Table 3** depicts the full design of the study.

**Table 3:** Full design of the two experiments.

	Phase 1		Phase 2		
			
Group	Training	Judgment	Training	Judgment	Question
Positive Outcome	Left-wing contingency (^∗^)	Judgment Phase 1	Right-wing contingency (^∗^)	Judgment Phase 2	Political orientation question
Negative Outcome					


*^∗^The order of Phases 1 and 2 was randomly assigned to each participant. They only differ in the instructional framing (government described as left- vs. right-wing).*

Immediately after providing a judgment for the second contingency training phase, participants answered some demographical questions: gender, age, educational level, and, crucially, a political self-positioning question. This question was worded exactly as presented in the official surveys by the Spanish National Centre for Sociological Studies (CIS): “When talking about politics, the words ‘left’ and ‘right’ are commonly used. Where would you place yourself on a scale from 0 to 10, where 0 represents the extreme left pole, and 10 represents the extreme right pole?” (Centro de Investigaciones Sociológicas, 2016). In sum, our measure of political positioning was carried out so that higher values (up to 10) are interpreted as positions leaned to the right (up to the extreme right pole), while lower values (up to 0) are interpreted as positions leaned to the left (up to the extreme left pole), and values close to 5 represent the political center.

### Results

#### Political Self-Positioning

**FIGURE 3:**
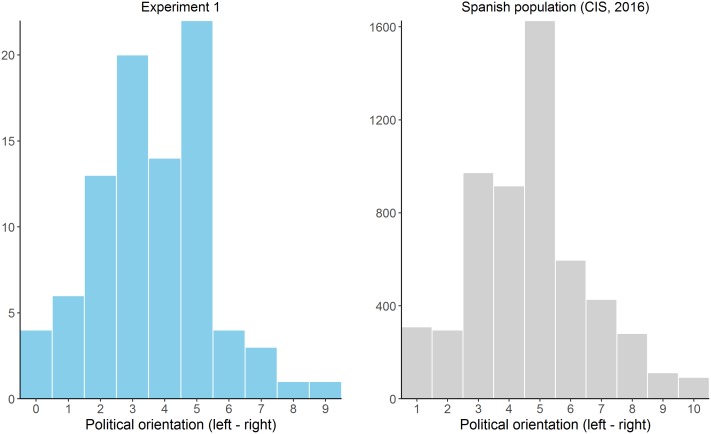
Histogram showing the distribution of the political orientation answers in Experiment 1 **(Left)** compared to a large-scale survey collected in the same population **(Right)**.

**Figure 3** (left panel) depicts a histogram of the answers given to the political self-positioning question. The distribution is slightly asymmetrical, with proportionally more participants in the left-part of the scale (*M* = 3.64, *SD* = 1.81, *Mdn* = 4.00), and no participants answering on the right extreme (i.e., 10). This is in line with previous observations in the Spanish population and is characteristic of well-educated participants such as ours (Centro de Investigaciones Sociológicas, 2016). To facilitate the comparison between our data and these previously observed trends, we also include the histogram corresponding to one official survey carried out in 2016 (**Figure 3**, right panel, *N* = 5,630 respondents), but note that in this national survey the political self-placement is assessed on a scale from 1 to 10. However, although this asymmetrical distribution is typical for our population, it posits a challenge for our design, given that we predict differences at the extremes of the political spectrum.

#### Judgments

**FIGURE 4:**
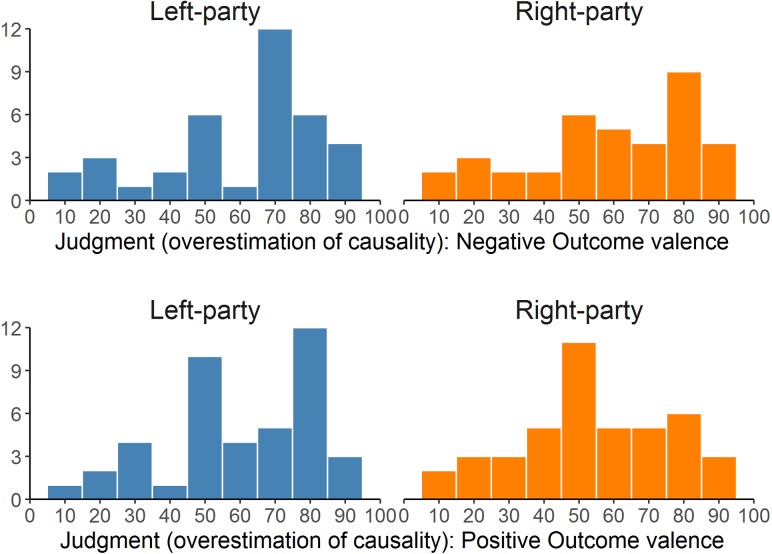
Distributions of the judgments given for the two parties (left- and right-wing) in the two outcome valence groups (positive vs. negative) in Experiment 1. The higher the judgment, the stronger the overestimation of causality (causal illusion).

In **Table 4**, we collect the descriptive statistics for the judgments given to the two contingency training phases, separated by outcome-valence group. Their distribution is also showed in **Figure 4**. Our prediction was that these judgments should vary as a function of: (a) the instructional framing (left-party vs. right-party), (b) the outcome-valence group (positive vs. negative), and (c) the political orientation.

**Table 4:** Descriptive statistics of the judgments (Experiment 1).

	Left-party phase		Right-party phase	
		
Outcome valence group	*M*	*SD*	*M*	*SD*
Positive Outcome (*n* = 42)	59.02	25.58	51.00	25.57
Negative Outcome (*n* = 46)	53.74	29.15	51.50	29.18


Therefore, we conducted a mixed 2 (instructional framing) × 2 (outcome valence) analysis of covariance (ANCOVA), introducing the political orientation scores as a covariate in the model (see **Table 5**). The expected three-way interaction was found significant. This interaction was then explored by examining each outcome-valence group.

**Table 5:** Results of the mixed ANCOVA on the judgments (Experiment 1).

Source	*F*^∗^	*p*	$\eta_{p}^{2}$
Framing	2.567	0.113	0.030
Framing × Valence	6.920	0.010	0.076
Framing × Political orientation	0.355	0.553	0.004
Framing × Political orientation × Valence	5.279	0.024	0.059
Valence	1.376	0.244	0.016
Political orientation	0.720	0.399	0.008
Valence × Political orientation	1.308	0.256	0.015


*^∗^Degrees of freedom: 1, 84.*

**FIGURE 5:**
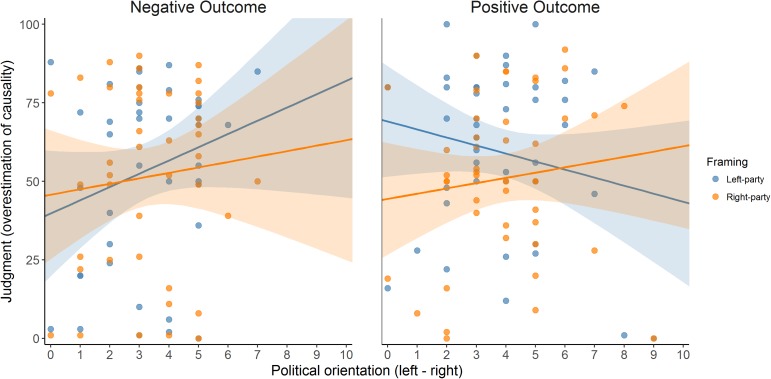
Judgments given to the two instructional framing conditions (Left- vs. Right-wing party) in the two outcome-valence groups (Positive vs. Negative Outcome), as a function of political orientation (*x*-axis), in Experiment 1. Since the actual contingency is actually zero, judgments can be interpreted as the degree of the overestimation of causality (causal illusion).

In the Positive Outcome group (**Figure 5**, right panel), the expected pattern was found. In this group, the outcome presented during the contingency training phases was positive (i.e., a desirable one). Consequently, participants whose political orientation was leaned to the left tended to give higher judgment to the left-wing party scenario than they did to the right-wing party scenario, despite both presenting actually the same contingency information. This tendency disappeared as participants positioned more to the right side of the political positioning scale (**Table 6**). Thus, our main prediction (that a participant would judge exactly the same information differently depending on the instructional framing, and that this difference would depend on the political positioning) was so far in line with the data.

**Table 6:** Results of the mixed ANCOVA on the judgments (Experiment 1) within each outcome valence group.

Positive Outcome group	*F*^∗^	*p*	$\eta_{p}^{2}$
Framing	7.254	0.010	0.142
Political orientation	0.066	0.798	0.001
Framing × Political orientation	4.048	0.050	0.084

**Negative Outcome group**	***F*^∗∗^**	***p***	**$\eta_{p}^{2}$**

Framing	0.716	0.403	0.018
Political orientation	1.385	0.246	0.033
Framing × Political orientation	1.688	0.201	0.040


*^∗^Degrees of freedom: 1, 44. ^∗∗^Degrees of freedom: 1, 40.*

Additionally, we conducted simple slope analyses to further study this result. To this end, we first computed difference scores to capture the within-participant effect of framing on the judgments (i.e., the judgment given to the left-wing party scenario minus the judgment given to the right-wing party scenario). Then, we regressed these difference scores on the political orientation score, after centering it on one point of the left-side of the scale (i.e., 1) and on one point of the right side of the scale (i.e., 9), two points that are symmetrical to the midpoint of the scale. By testing the intercepts of the regression lines on these two points, we can assess the extent to which participants on the ideological left and on the ideological right judge differently the information depending on the framing scenario. Thus, in the Positive Outcome group, the intercept for left-wing participants (political orientation = 1) was significantly positive, *B* = 20.431, *t*(44) = 2.794, *p* = 0.008, which means that their judgment in the left-wing party scenario was higher than in the right-wing party scenario. The opposite pattern (i.e., negative intercept) was expected for right-wing participants (ideological score = 9), although in this case the intercept was not significantly different from zero, *B* = -13.396, *t*(44) = -1.181, *p* = 0.244. This means that we found only partial support for our predictions, as participants in the right side of the political spectrum did not completely reverse the bias depending on the instructional framing. This result could be explained by the relatively small number of right-wing participants in our sample and invites to consider the need for a replication study.

The Negative Outcome group produced less clear results (**Table 6**, also **Figure 5**, left panel). Our prediction was the same as in the Positive Outcome group but reversed: As the outcome in this group is undesired, participants should tend to give higher judgments when they judge a party of the opposite pole of the spectrum (i.e., right-wing party for participants in the left pole, and vice versa). This was not what we found. As **Figure 5** (left panel) suggests, participants seemed insensitive to the instructional framing manipulation (i.e., left- vs. right-wing party). In fact, when we analyzed this group, we found no significant effect of either instructional framing, ideological positioning, or their interaction (all *p*s > 0.20). On the other hand, one must bear in mind the asymmetry of the ideological scores distribution, which means that we had few data points in the right-wing pole of the spectrum in this group (maximum ideological positioning score was 7 in the Negative Outcome group compared to 9 in the Positive Outcome group). Finally, the simple slopes analyses (conducted in the same way as in the Positive Outcome group) revealed that participants with either left-wing (i.e., 1) or right-wing (i.e., 9) political scores tended not to show differences between the two framing conditions because the intercepts of the difference scores in these two points were not significantly different from zero: *B* = -3.505, *t*(40) = -0.644, *p* = 0.523 when political score is 1 and *B* = 16.388, *t*(40) = 1.445, *p* = 0.156 when political score is 9.

## Experiment 2

The results of Experiment 1, obtained in a sample of Spanish participants, were partially in line with our predictions. When the task involved a positive-valenced outcome, we found the expected interaction between political orientation and instructional framing, although the effect was small. On the other hand, simple slope analyses revealed that the prediction was fulfilled concerning left-wing participants but not concerning right-wing participants (i.e., the intercept was not significantly different from zero when tested on a political orientation score corresponding to the right-hand of the scale). This could be due to the lack of right-wing participants in our sample: as shown in **Figure 3**, our sample was asymmetrical along the political orientation dimension, with proportionally more left-wing participants than ideologically centered and right-wing participants. Additionally, the group in which the outcome was of negative valence did not produce significant results. Therefore, we decided to conduct a replication study on a different population, with a larger sample, and trying to reach participants from the whole political orientation dimension to ensure that our conclusions hold firm.

### Method

#### Participants and Apparatus

We conducted an *a priori* power analysis protocol with G^∗^Power 3 (Faul et al., 2007). According to this analysis, to observe the main result from Experiment 1 (i.e., the interaction between political orientation and framing on the judgments in the Positive Outcome group), which was 0.084, with a power of 0.80, we need a sample of 93 participants in each group. Thus, we determined that our sample would be of about 200 participants (for two groups). A sensitivity analysis reveals that with this sample size and design, we can detect effects as small as *f* = 0.199 with 80% power.

The sample was collected online through the Prolific Academic website. Each participant received £1.50 for his/her time. A total of 195 participants produced valid data (i.e., data from five participants were not recorded due to technical error). The computer program randomly assigned them to each of the two groups: 100 were assigned to the Positive Outcome group and 95 to the Negative Outcome group. All participants were UK citizens (116 women and 79 men), with mean age of 36.94 years (*SD* = 12.746). Their education level is described in **Table 7**.

**Table 7:** Distribution of participants per education level (Experiment 2).

Education level	*n*
No studies	1
Primary education	1
Secondary education	35
Vocational training	17
University studies	100
Postgraduate studies	41


#### Procedure

The procedure was identical to that of Experiment 1, except for the translation of all instructions and texts into English (this task was conducted by a professional translator). Additionally, the participants were pre-screened through the Prolific Academic website, so that only those who had self-defined as “Left” or “Right” in the political UK scenario were allowed to participate. Regardless of this answer (which was unknown to the researchers), we assessed political orientation in our study through the same self-placement scale as in Experiment 1.

### Results

#### Political Self-Positioning

**FIGURE 6:**
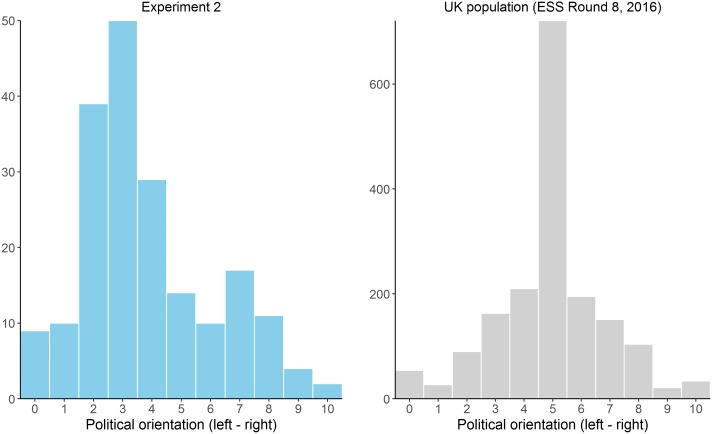
Histogram showing the distribution of the political orientation answers in Experiment 2 **(Left)** compared with a large survey on the UK population **(Right)**.

The histogram in **Figure 6** (left) depicts the distribution of our sample along the political orientation dimension. As in Experiment 1, the distribution contains proportionally more participants positioning themselves on the left-side of the spectrum (*M* = 3.831, *SD* = 2.258) than on the right side. Fortunately, we were able to reach several participants on the extreme right pole in this sample (six participants answered 9 or 10 on this scale). Additionally, the right-hand panel in **Figure 6** depicts the distribution of a large survey (*N* = 1770) conducted in the United Kingdom by the European Social Survey (European Social Survey, 2016), which is included for comparative purposes. As we can see, our sample does not mirror the typical political orientation distribution in the United Kingdom (we have proportionally more left-wing participants and fewer in the political center, while the UK population seems to be symmetrical along this spectrum). This can be due to a number of reasons, including our pre-screening stage (see previous section), or the likely possibility that the population of British participants in online studies does not represent well the whole British population (e.g., the distribution by level of education in **Table 7** does not match that of the whole UK population).

#### Judgments

**FIGURE 7:**
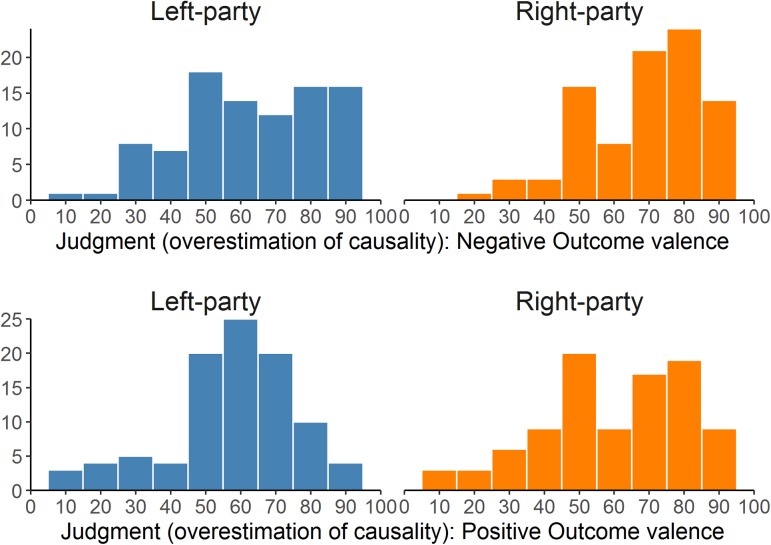
Distributions of the judgments given for the two parties (left- and right-wing) in the two outcome valence groups (positive vs. negative) in Experiment 2. The higher the judgment, the stronger the overestimation of causality (causal illusion).

**FIGURE 8:**
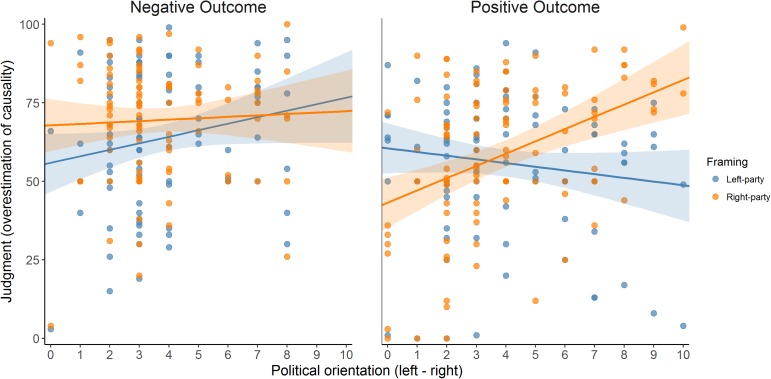
Judgments given to the two instructional framing conditions (Left- vs. Right-wing party) in the two outcome-valence groups (Positive vs. Negative Outcome), as a function of political orientation (*x*-axis), in Experiment 2. Since the actual contingency is actually zero, judgments can be interpreted as the degree of the overestimation of causality (causal illusion).

**Table 8** presents the descriptive statistics of the judgments in Experiment 2 (see also **Figure 7**, which depicts the distributions as histograms). As in the previous experiment, we conducted a mixed 2 (instructional framing) × 2 (outcome valence) ANCOVA, with political orientation as a covariate. The results can be seen in **Table 9**. Crucially, the expected three-way interaction was significant. This suggests that the interaction between political attitude and framing depends on the valence of the outcome. The scatterplots in **Figure 8** align with this impression.

**Table 8:** Descriptive statistics of the judgments (Experiment 2).

	Left-party phase		Right-party phase	
		
Outcome valence group	*M*	*SD*	*M*	*SD*
Positive Outcome (*n* = 100)	55.91	21.631	58.57	23.601
Negative Outcome (*n* = 95)	63.64	21.268	69.53	18.720


**Table 9:** Results of the mixed ANCOVA on the judgments (Experiment 2).

Source	*F*^∗^	*p*	$\eta_{p}^{2}$
Framing	0.780	0.378	0.004
Framing × Valence	22.990	<0.001	0.107
Framing × Political Orientation	6.162	0.014	0.031
Framing × Political Orientation × Valence	23.153	<0.001	0.108
Valence	3.739	0.055	0.019
Political orientation	4.942	0.027	0.025
Valence × Political Orientation	0.008	0.930	<0.001


*^∗^Degrees of freedom: 1, 191.*

Next, we conducted a mixed ANCOVA on the judgments of each outcome valence group, with political orientation and instructional framing as factors (**Table 10**). In the Positive Outcome valence group, the results were as expected: the two-way interaction indicates that political orientation predicts a different judgment depending on the framing condition. For left-wing participants, the left-wing party receives higher judgments than does the right-wing party, and this tendency reverses as participants’ self-position moves toward the right side on the political scale. As in Experiment 1, we conducted simple slope analyses by regressing the difference scores (within-participant difference between the judgments of the two framing scenarios) on the political positioning and tested the intercept in two points: one on the left (i.e., political orientation = 1) and one on the right (i.e., political orientation = 9). In the Positive Outcome group, the intercept was significantly positive for left-wing participants (i.e., political orientation = 1), *B* = 12.238, *t*(98) = 3.456, *p* = 0.001, and significantly negative for right-wing participants (i.e., political orientation = 9), *B* = -28.439, *t*(98) = -5.473, *p* < 0.001, which indicates that participants with a somewhat extreme-left political orientation score showed a tendency to produce higher judgments in the left-wing framing scenario than in the right-wing framing scenario, while the opposite pattern was found in participants with extreme-right political orientation score.

**Table 10:** Results of the mixed ANCOVA on the judgments (Experiment 2) within each outcome valence group.

Positive Outcome group	*F*^∗^	*p*	$\eta_{p}^{2}$
Framing	16.367	<0.001	0.143
Political orientation	3.523	0.064	0.035
Framing × Political orientation	30.614	<0.001	0.238

**Negative Outcome group**	***F*^∗∗^**	***p***	**$\eta_{p}^{2}$**

Framing	8.153	0.005	0.081
Political orientation	1.869	0.175	0.020
Framing × Political orientation	2.669	0.106	0.028


*^∗^Degrees of freedom: 1, 98. ^∗∗^Degrees of freedom: 1, 93.*

By contrast, in the Negative Outcome valence group, the expected interaction between political orientation and framing was not significant (the same happened in Experiment 1). The simple slope analyses are less useful in this situation but still indicate that left-wing participants (i.e., political orientation score = 1) gave higher judgments to the left-wing scenario as revealed by the significantly negative intercept, *B* = -10.311, *t*(93) = -3.095, *p* = 0.003, while right-wing participants (i.e., political orientation score = 9) did not show differences between the framing scenarios, since the intercept was non-significant, *B* = 2.679, *t*(93) = 0.479, *p* = 0.633.

## Discussion

As exposed in the Introduction, people are known to bias their causal learning under certain circumstances, leading to causal illusions, that is, to believe that a causal relationship exists when there is no actual contingency between the potential cause and the effect (Matute et al., 2015). The causal illusion could underlie many irrational beliefs and practices that are widespread, including pseudomedicine usage (Blanco et al., 2014; Yarritu et al., 2015), paranormal belief (Blanco et al., 2015), and social prejudice (Hamilton and Gifford, 1976; Kutzner et al., 2011). At the same time, the illusion has also been associated to well-being and optimism, as it is less prevalent in depressed people (Taylor and Brown, 1988; Blanco, 2017).

Previous research has identified various factors that modulate this bias from the observed frequencies of the events involved (Allan and Jenkins, 1983; Buehner and May, 2004) to individual differences such as mood (Alloy and Abramson, 1979; Msetfi et al., 2005; Blanco et al., 2012). Yet another factor that could modulate the illusion is previous knowledge and expectations (Yarritu and Matute, 2015). In line with this possibility, research on the related literature of “motivated cognition” (Kunda, 1990) suggests that people’s causal inferences can be either accurate or biased, depending on which outcome better fits previous beliefs, opinion, and worldview (Kahan et al., 2012). Thus, we take this argument further and propose that the causal illusion will be developed selectively to favor those conclusions that align with previous beliefs and ideology. In other words, if causal learning is a fundamental ability that underlies the acquisition of relevant parts of the knowledge that humans possess, including their worldviews and political beliefs, we suggest that the same ability could also be responsible for the preservation of inaccuracies in such knowledge because it contains biasing mechanisms that allow for the interpretation of ambiguous materials in a way that aligns with previous beliefs.

In our two studies, participants from two countries (Spain in Experiment 1, United Kingdom in Experiment 2) were exposed to contingency information corresponding to two causal scenarios, which differed only in the political orientation of the agent that carried out the actions: it was either a left-wing party or a right-wing party. The two scenarios were presented consecutively in randomly decided order. Except for the instructional framing, the contingency information was identical in the two phases and pointed to a null contingency (i.e., the potential cause and the effect were completely independent of each other). However, participants tended to show an illusion of causality (an overestimation of the null contingency) that was systematically stronger for one scenario than for the other. In addition, this difference was modulated by their political self-positioning, so that leftists judged the causal relationship between the left-wing party actions and the positive outcome stronger than the causal relationship between the right-wing party actions and the positive outcome. We expected to find the opposite pattern on right-wing participants, although this was only clear in Experiment 2, which was substantially better powered and included more right-wing ideological scores than did Experiment 1. In sum, our two experiments indicate that participants selectively developed stronger causal illusions when the potential existence of a causal relationship aligned with their previous beliefs and attitudes, and where more resistant to the illusion when the potential causal relationship would contradict such set of beliefs, at least when the outcome was positive.

This suggests that the causal illusion is a bias that appears selectively in the service of previously acquired causal schemata that are in some way meaningful for the participant’s worldview or belief system. These results were found in two different countries (Spain and United Kingdom), suggesting that they do not depend on particular features of the population. It is the first time, to our knowledge, that the causal illusion is characterized as a motivated mechanism to preserve previous held beliefs and worldview.

However, the expected association between the tendency to causal illusions and political attitudes (modulated by the instructional framing) was clear only in the group in which the outcome was described as positive. The result was much less clear in the Negative Outcome group. Although the tendency in the latter group was to increase judgments in the left-party phase more than in the right-party phase as ideological positioning shifts toward the right pole, the interaction was not significant. In principle, we were expecting to obtain the same results in the two outcome valence groups, only reversed depending on the valence. However, we found that when the outcomes were described in negative terms, the interactions failed to reach significance in the two experiments. In the literature of causal illusions, outcomes are usually of positive valence (e.g., something that the participant aims to produce, such as healing fictitious patients), and relatively few studies use negatively framed outcomes. Perhaps the mechanism that produces the illusion is dependent on, or sensitive to, the valence of the outcome (positive/negative), so that it becomes stronger in some conditions than in others, as suggested by previous research (Aeschleman et al., 2003; Bloom et al., 2007; Matute and Blanco, 2014). This might also explain why the literature covers mostly the setting that seemingly produces stronger effects (i.e., positive outcome).

There is some debate about the processes that originate the illusion. In particular, some have proposed that the bias appears in the moment of providing the judgment, rather than during the encoding phase, or training (Allan et al., 2005), although other support the opposite conclusion (Vadillo et al., 2016). Our current experiments were not designed to identify the process that produces the causal illusion or to isolate the moment in which it appears. Thus, although the exact nature or function of this causal illusion mechanism cannot be confirmed with our current results, what seems evident is the influence of previous beliefs on the modulation of the illusion.

There is one potential explanation for our results that is somewhat different from what we presented in the Introduction section. Overall, our results can be interpreted as participants judging differently the actions for the group with which they identify and the group against which they align (i.e., an ingroup/outgroup effect), assuming that left-wing participants see the left-wing party as their ingroup and the right-wing party as the outgroup. The implication is that our results should be observed in any experimental setting in which participants judge actions by their ingroup and outgroup, irrespective of whether the groups are framed in terms of political orientation or any other type of attitude or membership (e.g., attitudes toward ecology, country of residence, hobbies…). This interpretation does not affect our main conclusions (that the causal illusion can be used selectively to protect prior beliefs) but contribute to extend them to other situations that we have not tested. Still, studying the effect of political orientation is relevant because this attitude affects many important decisions taken at the individual and collective levels.

Additional conclusions can be drawn from our study. For example, we found that participants on both sides of the political spectrum exhibited (to some extent) the causal illusion in the situation that aligned with their prior beliefs. This result agrees with recent research indicating that ideologically driven biases are features of human nature in general and can be found in both leftists and rightists (Brandt et al., 2014) and is contrary to what has been previously suggested, that is, the prejudice gap hypothesis, which states that biases (but also prejudices and extreme opinions) are characteristic of the right (Chambers et al., 2013).

Most of the research on cognitive biases and political orientation has been carried out in the United States in which political positioning is more clearly polarized (e.g., between democrats and republicans). This allowed previous studies to dichotomize their sample into two main ideological groups with a set of largely homogenous prejudices and attitudes (e.g., most republicans hold similar beliefs about gun-control policies), hence simplifying their designs. This is not completely applicable to the case of most European countries, like Spain, where the political orientation cannot be clearly dichotomized and individuals hold a mixture of beliefs and prejudices that do not change uniformly across the political dimension (Centro de Investigaciones Sociológicas, 2015). As a consequence, we had to treat political orientation as a continuous variable, which complicated our interpretation of the results.

To sum up, on the light of our results, we propose that people interpret the causal information at their convenience and as a function of their previous attitudes and beliefs. Leftists and rightists not only disagree about their views on how the world should be and in their judgments about complex sets of information that are open to multiple interpretations. As our research shows, they can also provide substantially different interpretations even when they are exposed to identical data in its simplest and most objective form.

## Author Contributions

FB, BG-F, and HM conceptualized the study and reviewed the manuscript. FB programmed the experiment and wrote the draft. FB and HM analyzed the data.

## Conflict of Interest Statement

The authors declare that the research was conducted in the absence of any commercial or financial relationships that could be construed as a potential conflict of interest.
